# Determination of Johnson–Cook Constitutive and Failure Parameters for Cr20Ni80 Alloy Using an Experimental–Numerical Approach

**DOI:** 10.3390/ma19091909

**Published:** 2026-05-06

**Authors:** Zhi Li, Xuejin Yang, Kemin Zhou, Shaoyun Song, Meili Cao, Rui Li

**Affiliations:** 1 School of Mechanical Engineering, Wuhan Polytechnic University, Wuhan 430023, China; litchiworkemail@163.com (Z.L.);; 2 School of Power and Mechanical Engineering, Wuhan University, Wuhan 430072, China

**Keywords:** Cr20Ni80 alloy, constitutive model, failure model, parameter calibration

## Abstract

Accurate numerical simulation of Cr20Ni80 alloy processing relies on reliable constitutive and failure models. This study employs a comprehensive experimental–numerical approach to calibrate and validate the Johnson–Cook (J-C) parameters of Cr20Ni80 alloy under varying stress states and strain rates. Quasi-static tensile tests on smooth and notched specimens, alongside dynamic Split Hopkinson Tension Bar (SHTB) tests (1000–3000 s^−1^), were conducted. Pulse-shaping technology was employed, and dynamic force balance was verified to ensure the physical validity of the high-strain-rate data. The constitutive parameters (A=621.02 MPa,  B=543.20 MPa,  n=0.4564,  C=0.0141) were determined based on true stress–strain responses. Theoretical analysis confirms that the thermal softening effect caused by adiabatic heating can be neglected. Furthermore, the failure parameters ($D_{1}=-0.4300,{\;D}_{2}=2.6405,{\;D}_{3}=-0.7055$) were calibrated to capture the stress triaxiality effects ($R^{2}=0.978$). The parameter $D_{4}$ was iteratively calibrated using SHTB data from the 1000 s^−1^ and 3000 s^−1^ test conditions and validated using SHTB data from the 2000 s^−1^ test condition. The engineering stress–strain curves obtained from simulations using the calibrated parameters showed good agreement with experimental results, confirming the reliability of the calibrated parameters.

## 1. Introduction

Cr20Ni80 alloy is a nickel-chromium electric heating material with excellent macroscopic physical properties and thermal stability. It features a high melting point, stable resistivity, low coefficient of thermal expansion, and good oxidation resistance and corrosion resistance at high temperatures. It is widely used in extreme operating environments across industries such as aerospace, marine, chemical, and energy [1]. During the machining of Cr20Ni80 alloy, numerous nonlinear factors—including the strain-hardening, strain-rate sensitivity, and thermal response of the material, the geometric characteristics of the workpiece or specimen, and the contact interactions that govern load transfer and local stress distribution—come into play. These factors jointly lead to complex and varied stress–strain conditions during the forming process. Relying solely on traditional experimental analysis and empirical formulas not only makes calculations difficult but also makes it challenging to accurately describe this process [2,3,4,5,6]. Studying the machining and forming of Cr20Ni80 alloy through numerical simulation can clarify macroscopic deformation mechanisms, predict failure phenomena, and optimize process parameters to shorten development cycles and reduce costs. However, the reliability of numerical simulation depends entirely on the accuracy of its underlying constitutive and damage models. The Johnson–Cook (J-C) model is renowned for its mathematical simplicity and its ability to describe strain hardening, strain rate sensitivity, and thermal softening, making it one of the most widely used phenomenological models in the field of impact dynamics.

Previous studies on Cr20Ni80 alloy have mainly focused on deformation mechanisms and microstructural evolution under hot deformation, annealing, and tensile loading conditions. Dudova [7] investigated the deformation mechanisms of Cr20Ni80 alloy at elevated temperatures and pointed out that the available data still did not provide a complete picture of the physical processes controlling plastic flow. More recently, Zhang [8,9] reported the microstructural evolution and deformation behavior of Cr20Ni80 alloy during tensile deformation, highlighting the roles of twin boundaries, dislocation slip, grain rotation, and related strengthening mechanisms.

Meanwhile, the calibration of Johnson–Cook constitutive and failure parameters has been widely reported for engineering alloys through combined experimental and numerical approaches. Representative studies include the determination of J-C plasticity parameters for CoCrMo alloy, the step-by-step calibration of J-C constitutive and damage parameters for structural steel, and the identification of J-C material and failure parameters for armour steel and pipeline steel [10,11,12,13,14]. These studies demonstrate that the J-C model can provide a practical constitutive-damage description when supported by quasi-static tests, dynamic tests, and finite element validation.

Although the Cr20Ni80 alloy is now widely used in industrial applications, there are few studies in the literature on its dynamic mechanical behavior or on the calibration of its J-C constitutive parameters and failure parameters. Although alternative constitutive descriptions, such as modified Johnson–Cook, Arrhenius-type, or Hockett–Sherby-type laws, have also been proposed for thermomechanical deformation analysis, the classical J-C model still provides a practical balance between model simplicity, parameter identification, and implementation in commercial finite element software [15,16]. To address this research gap, this study employed a combined experimental and numerical simulation approach. Through tensile tests (on smooth and notched specimens) and Split Hopkinson Tension Bar (SHTB) experiments at varying high strain rates ($1000–3000\;s^{-1}$), the mechanical response of the alloy under various strain rate ranges was obtained. By processing and analyzing the experimental data using numerical simulation techniques, the J-C constitutive parameters and failure criteria for the Cr20Ni80 alloy were calibrated. The engineering stress–strain curves obtained from simulations using the calibrated parameters are in good agreement with the SHTB test data, validating the effectiveness of the parameters.

## 2. Johnson–Cook Constitutive Model

The J-C constitutive model is a widely adopted empirical model primarily used to describe the plastic flow behavior of metallic materials under various strain rates and thermal conditions [17,18]. Its mathematical expression is concise, decoupling strain hardening, strain rate sensitivity, and thermal softening effects, and it reliably predicts material behavior under large plastic deformation, dynamic impact, and high-temperature conditions. The J-C flow stress equation is expressed as follows [11]:

(1) $$\sigma_{eq}=\left(A+B\varepsilon_{p}^{n}\right)\left(1+C\ln{\dot{\varepsilon }}^{*}\right)\left(1-T^{*m}\right)$$

where $\sigma_{eq}$ denotes the equivalent flow stress, and *$\varepsilon_{p}$* is the equivalent plastic strain. The dimensionless strain rate is defined as ${\dot{\varepsilon }}^{*}=\dot{\varepsilon }/{\dot{\varepsilon }}_{0}$, with $\dot{\varepsilon }$ and ${\dot{\varepsilon }}_{0}$ being the current and reference strain rates. The homologous temperature is defined as $T^{*}=(T-T_{r})/(T_{m}-T_{r})$, where T is the working temperature, $T_{\;r}$ is the reference room temperature, and $T_{m}$ is the melting temperature of the material. Regarding the material constants to be calibrated: A represents the initial yield stress at the reference strain rate and temperature; B and n are the strain hardening coefficient and the strain hardening exponent, respectively; C indicates the strain-rate sensitivity coefficient; and m is the thermal softening exponent.

### 2.1. Determination of Parameters A*, B* and n

To systematically calibrate the J-C parameters, a decoupled step-by-step methodology was adopted. For quasi-static tensile tests conducted at the reference room temperature ($T=T_{r}$), the thermal softening effect is inherently eliminated ($T^{*}=0$), and when the quasi-static tests are performed exactly at the selected reference strain rate ($\dot{\varepsilon }={\dot{\varepsilon }}_{0},\;\;yielding\;\dot{\varepsilon^{*}}=1$), the strain-rate hardening term diminishes ($\ln\dot{\varepsilon^{*}}=0$). Consequently, Equation (1) simplifies to [11]:

(2) $$\sigma_{eq}=A+B\varepsilon_{p}^{n}$$

According to Equation (2), uniaxial tensile tests must be conducted at a low and constant crosshead speed to obtain data for determining parameters A, B and n.

#### 2.1.1. Tensile Testing of Smooth Round Bars

**Figure 1:**
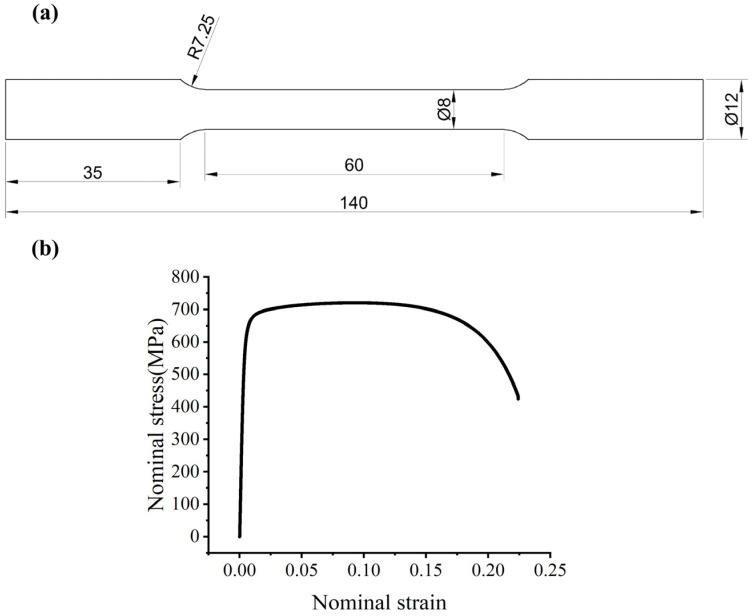
Geometry of the smooth tensile specimen and the corresponding engineering stress–strain response of Cr20Ni80 alloy: (**a**) specimen dimensions (unit: mm); (**b**) representative engineering stress–strain curve under quasi-static tension.

To determine the material’s constitutive relationship, quasi-static uniaxial tensile tests were conducted on smooth cylindrical specimens at room temperature. The chemical composition of the Cr20Ni80 alloy used in this study is shown in Table 1. The material was supplied by Baoshan Iron & Steel Co., Ltd. (Shanghai, China) as a commercial Cr20Ni80 round bar with a diameter of 12 mm. All smooth, notched, and SHTB specimens were machined from the as-received bar stock, and no additional heat treatment was applied during specimen preparation. Since the objective of the present work is the phenomenological calibration of the Johnson–Cook constitutive and failure parameters, no additional microstructural characterization was carried out in this study. The specimen dimensions are shown in Figure 1a. Testing was performed using a UTM400 series dual-column testing machine, with a constant crosshead displacement rate of 0.03 $mm\cdot s^{-1}$. The smooth tensile specimens were prepared in accordance with GB/T 228.1-2021 [19], and three repeated tests were conducted under the same conditions. An extensometer was employed to record the gauge section elongation of the smooth tensile specimen during the uniform deformation stage. The representative engineering stress–strain response of the Cr20Ni80 alloy is depicted in Figure 1b. After yielding, the flow stress continues to increase with increasing strain before the onset of necking, indicating a clear strain-hardening tendency. This tendency is consistent with previous tensile and deformation studies on Cr20Ni80 and related nickel-based alloys, in which strain hardening associated with dislocation activity and microstructural evolution has also been reported [7,8,9]. 

#### 2.1.2. Determination of Parameters A, B and n

**Figure 2:**
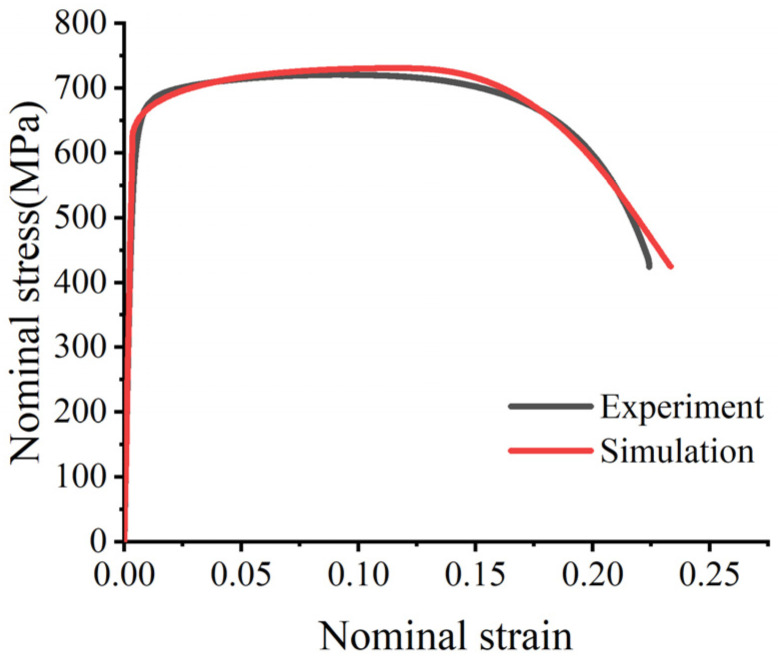
Comparison between the experimental engineering stress–strain response and the curve predicted by the calibrated J-C constitutive model for the smooth tensile specimen.

To accurately calibrate the baseline J-C flow parameters, the obtained engineering stress–strain data from the smooth bar tests were essentially converted into true stress and true plastic strain curves prior to the onset of macroscopic necking. Based on the applied crosshead speed and the initial gauge length of the specimen, the quasi-static reference strain rate was determined to be $\dot{\varepsilon_{0}}=0.5\times 10^{-3}\;s^{-1}$. In strict accordance with the standard 0.2% offset plastic strain criterion on the true stress–strain curve, the initial quasi-static yield stress parameter was unambiguously identified as A = 621.02 MPa. Subsequently, the true plastic flow behavior beyond the initial yield point was fitted using Equation (2) to determine the strain hardening coefficient B = 543.20 MPa and the strain hardening exponent n = 0.4564. The comparison between the experimental and simulated engineering stress–strain curves is shown in Figure 2.

### 2.2. Determination of Parameter C

At the exact onset of macroscopic yield, the accumulated equivalent plastic strain is assumed to be zero (ε= 0). Consequently, the strain hardening term in Equation (2) is eliminated, and the constitutive relationship simplifies to [11]:

(3) $$\sigma_{eq}=A\left(1+C\ln{\dot{\varepsilon }}^{*}\right)$$

With the quasi-static yield strength (A) successfully determined, the strain-rate sensitivity parameter (C) can be explicitly calibrated using representative dynamic flow stress data obtained from high-strain-rate experiments.

#### 2.2.1. Dynamic Split Hopkinson Tension Bar (SHTB) Testing

**Figure 3:**
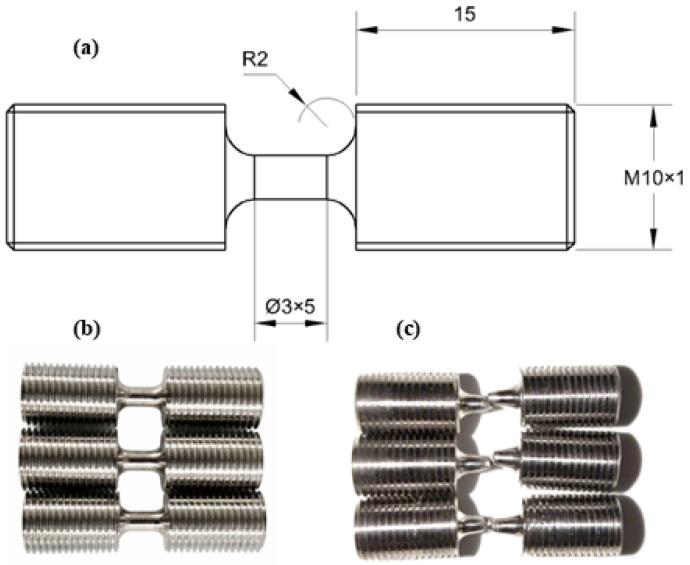
Geometry of the SHTB specimen and representative appearances before and after dynamic tensile testing: (**a**) specimen dimensions (unit: mm); (**b**) undeformed specimen; (**c**) representative fractured specimens after testing at different strain rates.

To characterize the dynamic mechanical response of the Cr20Ni80 alloy, high-strain-rate tensile tests were conducted using a Split Hopkinson Tension Bar (SHTB) apparatus at room temperature, which has emerged as the most extensively employed technique for experimentally assessing materials subjected to high strain rates ranging from $10^{2}$ to $10^{4}\;s^{-1}$ [21,22,23,24,25]. The specimen dimensions for dynamic testing are detailed in Figure 3a. The SHTB specimen geometry was designed with reference to GB/T 30069.1-2013 [26] for high-strain-rate tensile testing using an elastic-bar-type system.

Tests were executed at three representative nominal strain rates: $1000{\;s}^{-1}$, $2000\;s^{-1}$, and $3000{\;s}^{-1}$. For the present parameter calibration, one valid test was used at each nominal strain-rate level. A comparison between the original specimen and the deformed specimens post-testing is illustrated in Figure 3b,c.

**Figure 4:**
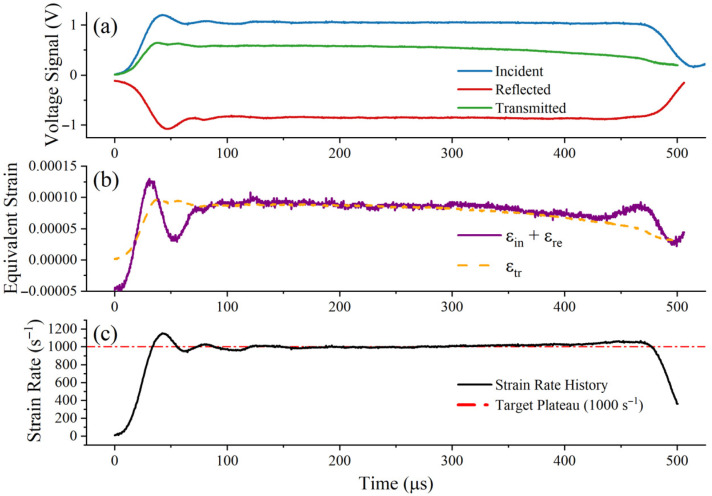
Verification of the SHTB test at 1000 s^−1^: (**a**) raw incident, reflected, and transmitted signals; (**b**) dynamic force equilibrium verification by comparing the transmitted signal with the sum of the incident and reflected signals; (**c**) strain-rate history showing the approximately constant strain-rate plateau during the main deformation stage.

**Figure 5:**
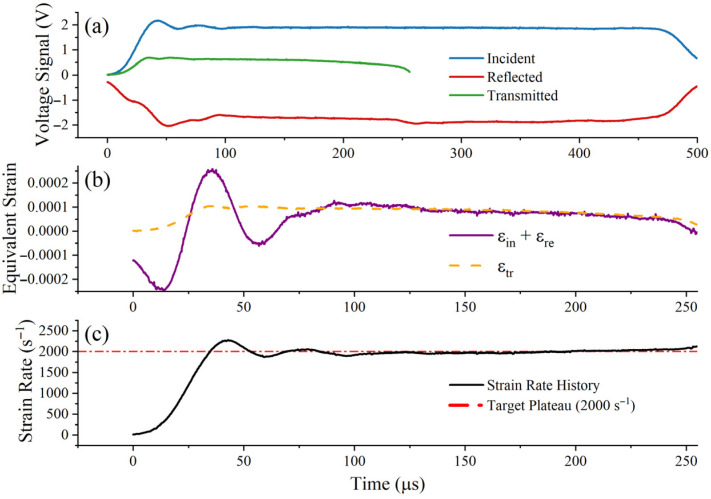
Verification of the SHTB test at 2000 s^−1^: (**a**) raw incident, reflected, and transmitted signals; (**b**) dynamic force equilibrium verification by comparing the transmitted signal with the sum of the incident and reflected signals; (**c**) strain-rate history showing the approximately constant strain-rate plateau during the main deformation stage.

**Figure 6:**
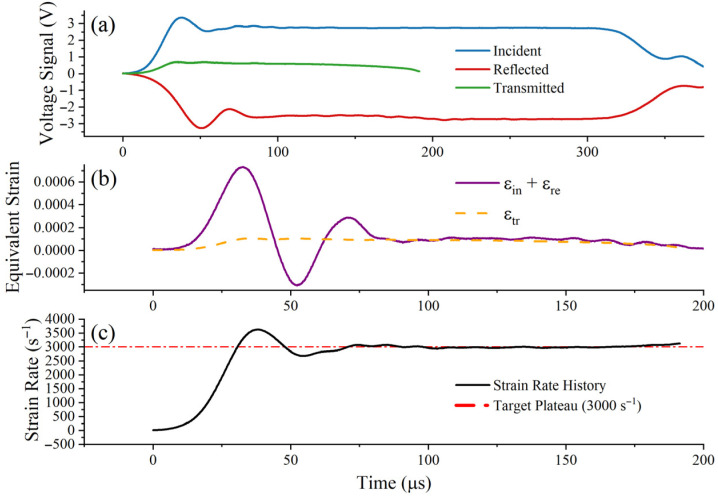
Verification of the SHTB test at 3000 s^−1^: (**a**) raw incident, reflected, and transmitted signals; (**b**) dynamic force equilibrium verification by comparing the transmitted signal with the sum of the incident and reflected signals; (**c**) strain-rate history showing the approximately constant strain-rate plateau during the main deformation stage.

To ensure the physical fidelity of the extracted dynamic data, a thin copper pulse shaper was attached to the impactor-incident bar interface [27]. Pulse-shaping technology smooths the rising edge of the incident wave and mitigates severe high-frequency oscillations caused by inertia. Figure 4, Figure 5 and Figure 6 show the raw voltage signals and validate the dynamic force balance and constant strain rate of the experiment. In these figures, the incident, reflected, and transmitted signals are presented separately, and the approximately constant strain-rate plateau can be observed during the main deformation stage [28]. At all strain rates, the sum of the incident and reflected strains ($\varepsilon_{i}+\varepsilon_{r}$) exhibits good agreement with the transmitted strain ($\varepsilon_{t}$) within the plastic deformation window, confirming that a uniform stress state has been achieved at both ends of the specimen; simultaneously, the strain rate remains stable at the target plateau.

#### 2.2.2. Determination of Parameter C and Adiabatic Heating Analysis

**Figure 7:**
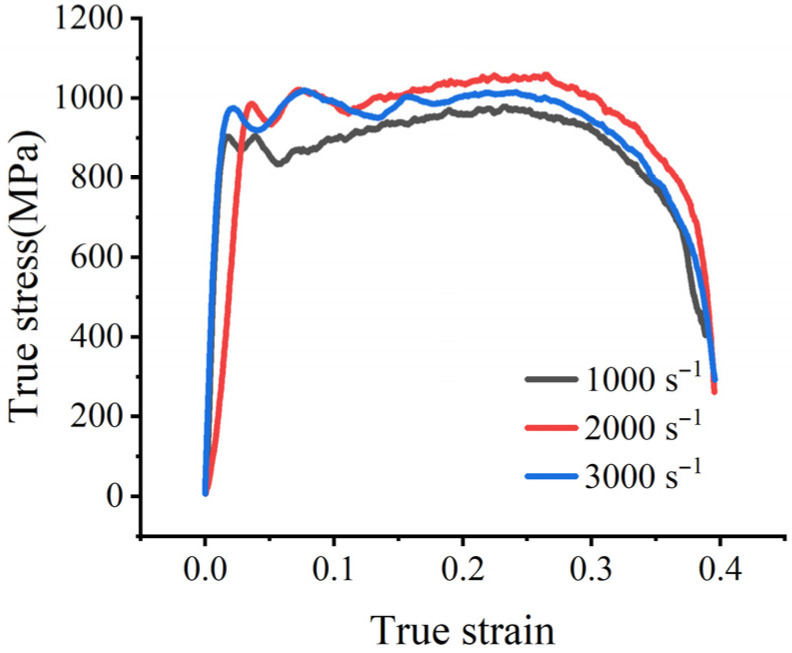
Dynamic stress–strain responses of Cr20Ni80 alloy obtained from SHTB tests at different strain rates. Only the data prior to the onset of necking were used for extracting the representative dynamic flow stress.

The stress–strain curve obtained after converting and processing the electrical signals recorded during the experiment is shown in Figure 7. Only the data prior to the onset of necking were used for extracting the representative dynamic flow stress, and the post-necking segment was not used for parameter identification. Under dynamic loading conditions, it is difficult to determine the initial yield point directly because the early part of the response is affected by inertial oscillations. As shown by the strain-rate histories in Figure 4, Figure 5 and Figure 6, after the plastic strain reaches 5%, the specimen has already entered the stable constant-strain-rate stage, while the response still remains prior to the onset of necking. Therefore, in this study, the true stress at 5% plastic strain was adopted as a representative dynamic flow stress for calibrating parameter C, rather than using the oscillatory initial portion of the curve. A similar treatment has also been reported in the literature for determining the strain-rate parameter of the Johnson–Cook model [10]. Table 2 shows the relationship between the strain rate and the extracted representative dynamic flow stress.

**Figure 8:**
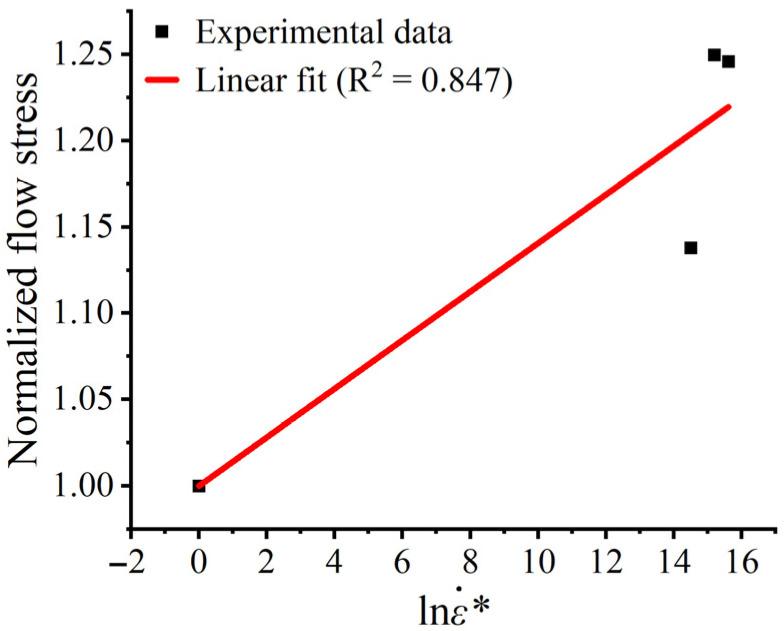
Linear fitting of the normalized dynamic flow stress as a function of the logarithm of the dimensionless strain rate for determining the strain-rate sensitivity parameter C.

At extremely high strain rates (3000 s^−1^), a portion of the plastic work is converted into heat, which may result in an adiabatic temperature rise (ΔT). According to the Taylor-Quinney empirical law [29], ΔT can be estimated by $\Delta T=\left(\beta /\rho C_{p}\right)\int \sigma d\varepsilon$, where the Taylor-Quinney coefficient is β= 0.9, the density is $\rho =8400\;{\mathrm{kg}/m}^{3}$, and the specific heat capacity is $C_{p}=440\;J/(\mathrm{kg}\cdot K)$. Integrating the actual stress–strain curve ($3000\;s^{-1}$) yields a maximum plastic work of *$380.25\;MJ/m^{3}$*, and the estimated adiabatic temperature rise is approximately 92.6 °C.The melting point of the Cr20Ni80 alloy is $T_{m}\approx 1400\;{}^{\circ}C$. Calculations yield $T^{*}\approx 0.067$, so the thermal softening effect can be neglected. Since the estimated adiabatic temperature rise is linearly proportional to the Taylor–Quinney coefficient, a moderate variation in β would not change the conclusion that $T^{*}$ remains low and that thermal softening has only a limited influence under the present test conditions. Based on Equation (3), the relationship between the normalized dynamic flow stress and the logarithm of the dimensionless strain rate was linearly fitted, as shown in Figure 8. According to the slope of the fitted line, the strain-rate strengthening coefficient was determined to be C = 0.0141, with a coefficient of determination of $R^{2}=0.847$.

Based on the systematic experiments and numerical calibration procedures, the obtained J-C constitutive model parameters for the Cr20Ni80 alloy at room temperature are shown in Table 3.

## 3. Johnson–Cook Failure Model

Ductile fracture failure often occurs in metal materials during large plastic deformation. These complex failure mechanisms are deeply influenced by stress state and show obvious sensitivity to strain rate and temperature. The classic J-C failure model is a phenomenological continuum damage criterion widely implemented in commercial finite element software for predicting ductile fracture [12,30,31,32,33,34]. The classic J-C model primarily focuses on the influence of stress triaxiality, which dominates void nucleation and coalescence under tensile loading conditions. Based on the cumulative damage law, the damage parameter D is defined as follows [17]:

(4) $$D=\sum \frac{\Delta \varepsilon }{\varepsilon_{f}}$$

where Δε is the increment of equivalent plastic strain within a single integration time step, and $\varepsilon_{f}$ denotes the equivalent fracture strain under the current state. Fracture is initiated when D reaches 1.0. The general expression for the J-C fracture strain $\varepsilon_{f}$ is given by [11]:

(5) $$\varepsilon_{f}=\left[D_{1}+D_{2}\exp\left(D_{3}\sigma^{*}\right)\right]\left[1+D_{4}\ln{\dot{\varepsilon }}^{*}\right]\left[1+D_{5}T^{*}\right]$$

in which $D_{1}$ to $D_{5}$ are material failure parameters. The stress triaxiality is defined as $\sigma^{*}=\sigma_{m}/\sigma_{eq}$, where $\sigma_{m}$ represents the hydrostatic stress and $\sigma_{eq}$ is the von Mises equivalent stress.

### 3.1. Determination of Parameters $D_{1}$ *, $D_{2}$, and $D_{3}$*

Under quasi-static loading conditions at the reference room temperature, the influences of strain rate and adiabatic heating are inherently eliminated ($\ln\dot{\varepsilon^{*}}=0$ and $T^{*}=0$). Consequently, the general failure equation simplifies to capture the pure effect of stress triaxiality [11]:

(6) $$\varepsilon_{f}=D_{1}+D_{2}\exp\left(D_{3}\sigma^{*}\right)$$

#### 3.1.1. Quasi-Static Tensile Tests of Notched Bars

**Figure 9:**
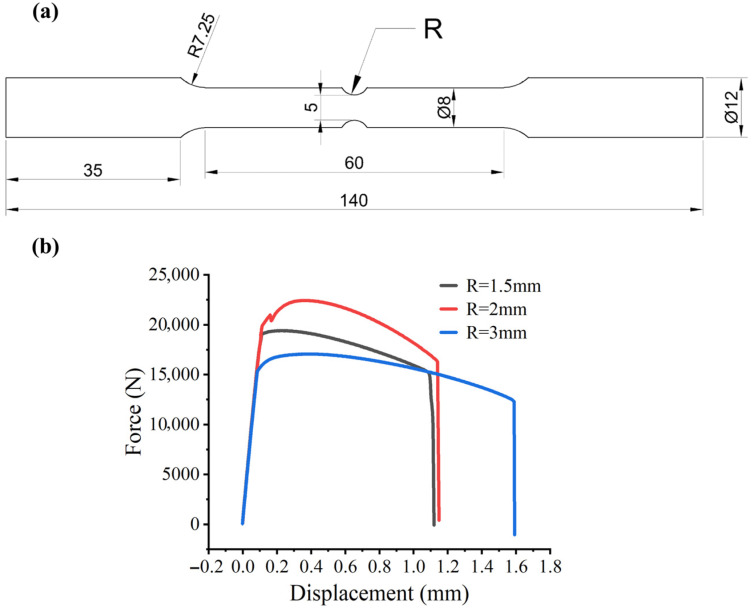
Geometry of the notched tensile specimens and the corresponding experimental load–displacement responses: (**a**) dimensions of specimens with different notch radii (unit: mm); (**b**) load–displacement curves obtained from quasi-static tensile tests.

To determine the parameters $D_{1}$, $D_{2}$, and $D_{3}$, it is imperative to acquire fracture strain data under varying stress states. Stress triaxiality ($\sigma^{*}$) serves as the critical parameter quantifying the complex stress state that governs ductile fracture. In order to systematically study its effect on fracture strain of Cr20Ni80 alloy, quasi-static tensile tests were carried out on notched round bar samples at room temperature. By adjusting the geometric shape of the notch, different initial stress triaxiality levels can be effectively realized. Consequently, three distinct notch geometries were designed, featuring notch radii of 1.5 mm, 2.0 mm, and 3.0 mm. No dedicated standard was identified for room-temperature quasi-static notched round-bar tensile specimens used for stress-triaxiality-dependent calibration of the Johnson–Cook failure model. Therefore, the specimen dimensions were designed with reference to published studies on this type of calibration [10,11,12]. One quasi-static tensile test was conducted for each notch radius. The specific dimensions of the notched specimens are shown in Figure 9a, while the corresponding macroscopic load–displacement relationship is presented in Figure 9b.

#### 3.1.2. Determination of Parameters $D_{1},D_{2}$, and $D_{3}$

Bridgman’s analysis [35] indicates that the initial stress triaxiality at the center of the notch tensile specimen can be estimated using the empirical relationship $\sigma^{*}=1/3+\ln\left(1+a/2R\right)$, where a is the minimum cross-sectional radius and R is the radius of the notch profile. However, this analysis makes an approximate assumption of a rigid-ideal plastic material and cannot accurately describe the drastic evolution of stress triaxiality during local necking and large deformation before the fracture occurs.

To overcome the limitation of this analysis and obtain accurate stress triaxiality and fracture strain history, a numerical extraction methodology was employed in this study. By utilizing fully calibrated J-C constitutive parameters, an explicit finite element model of the notch specimen was constructed in Abaqus 2023 (Dassault Systèmes Simulia Corp., Johnston, RI, USA).. To ensure computational reliability and alleviate mesh dependency issues during severe localization, 8-node linear brick, reduced integration elements with hourglass control (C3D8R) were adopted. Based on preliminary mesh sensitivity verifications, a refined characteristic element size of 0.5 mm was assigned to the critical notch region. The boundary conditions and loading mode in the simulation were defined to reproduce the corresponding quasi-static tensile test.

**Figure 10:**
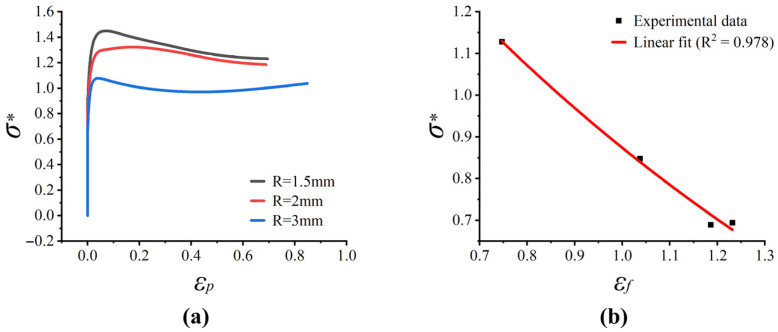
Numerically extracted fracture data and fitted fracture locus for Cr20Ni80 alloy under different stress triaxiality states: (**a**) equivalent fracture strain data obtained from FE-assisted analysis of notched specimens; (**b**) exponential fitting of fracture strain as a function of stress triaxiality based on the J-C failure model.

Ductile fracture typically initiates at the geometric center of the minimum cross-section, where the macroscopic stress triaxiality reaches its maximum. Thus, the temporal evolution of stress triaxiality was explicitly extracted from the central element. Theoretically, the macroscopic fracture strain ($\varepsilon_{f}$) can be determined by the geometric reduction in the minimum cross-section, expressed as $\varepsilon_{f}=\ln\left(A_{0}/A_{f}\right)=2\ln\left(r_{0}/r_{f}\right)$, where $A_{0}$ ($r_{0}$) and $A_{f}$ ($r_{f}$) are the initial and fractured cross-sectional areas (radii), respectively [11]. However, direct experimental measurement of $A_{f}$ introduces significant inaccuracies due to the severe distortion, non-circularity, and surface irregularities of the post-mortem fracture surfaces. Given that the established FE models accurately capture the macroscopic load–displacement responses of the notched specimens, the temporal evolution of the minimum cross-sectional area was extracted from the numerical simulations. Consequently, the equivalent fracture strains were calculated based on the simulated geometric reduction at the exact instant of macroscopic crack initiation. The resulting equivalent plastic strains extracted from the numerical simulations under varying stress triaxiality states are plotted in Figure 10a.

Subsequently, these data points are fitted by using simplified failure equations, and the failure parameters of baseline materials are obtained: $D_{1}=-0.4300$, $D_{2}=$2.6405, and $D_{3}=-0.70545$. The fitted exponential curve (Figure 10b) exhibits an exceptionally high coefficient of determination ($R^{2}=0.978$). This strong correlation proves that the phenomenological exponential decay relation proposed by the J-C failure model accurately captures the macroscopic fracture behavior of Cr20Ni80 alloy under different stress triaxiality states, which lays a foundation for the subsequent dynamic damage prediction.

### 3.2. Determination of Parameter $D_{4}$

**Figure 11:**
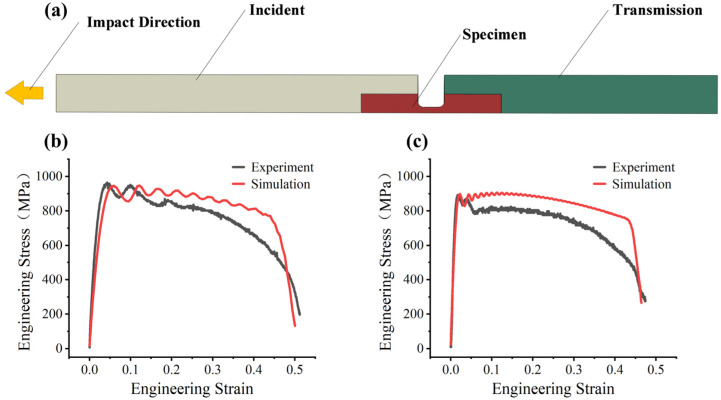
Finite element model of the SHTB test and experimental–numerical comparisons used for inverse calibration of parameter $D_{4}$: (**a**) simplified three-dimensional FE model; (**b**) comparison between experimental and simulated engineering stress–strain curves at 1000 s^−1^; (**c**) comparison between experimental and simulated engineering stress–strain curves at 3000 s^−1^.

During SHTB tests, the transmission bar’s rebound causes deformation at the fracture surface. Due to the complex geometry of the fracture surface after SHTB testing, direct measurement of $A_{f}$ introduces significant errors. Therefore, a method combining SHTB experiments with finite element simulations for inverse calibration was adopted to determine the parameter $D_{4}$. The SHTB tests were replicated in Abaqus/Explicit 2023 by creating and configuring a corresponding simulation model. The three-dimensional model used for simulation after reasonable simplification is shown in Figure 11a. All dimensions of each part match those of the experimental setup. The experimentally measured loading pulse and the corresponding boundary constraints were directly adopted in the simulation. Fine meshing was applied to the impact zone, bar-specimen contact regions, and parallel section of the specimen, while coarse meshing was adopted elsewhere. Local mesh refinement in these regions was introduced to improve the accuracy of stress-wave transmission and local deformation prediction. The C3D8R element was adopted, and the loading conditions and boundary constraints were fully adopted from the experiments. The calibrated J-C constitutive parameters and partial damage parameters were assigned to the specimen model. The parameter $D_{4}$ was assigned an initial value. After each simulation, the stress–strain curves were extracted and compared with the experimental data (1000 s^−1^ and 3000 s^−1^), and $D_{4}$ was adjusted iteratively. When the simulated curves showed significant agreement with the experimental curves, the optimal value was determined. After multiple calculations, $D_{4}=-0.0355$ was found to provide the best fit between the simulation and experimental results. Figure 11b,c show the simulation results obtained using this value.

**Figure 12:**
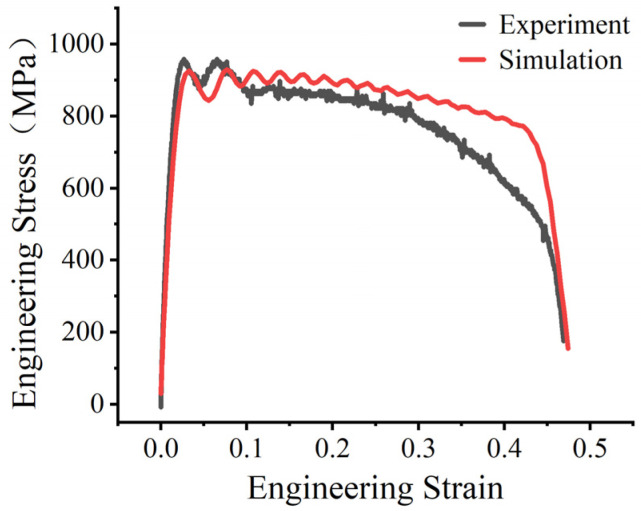
Comparison between the experimental and simulated engineering stress–strain curves at 2000 s^−1^ for validation of the calibrated J-C constitutive and failure parameters.

To verify the reliability of all calibrated parameters, a simulation of the SHTB test under a strain rate of $2000\;s^{-1}$ was performed using the calibrated parameters; the results are shown in Figure 12. Due to the inherent stress wave dispersion in the SHTB test and the decoupled nature of the J-C plasticity model, slight deviations were observed during the initial plastic flow stage. Nevertheless, the overall trend of the experimental response was reproduced reasonably well by the simulation, including the main deformation stage and the subsequent stress drop associated with fracture, which supports the validity of the calibrated parameter set. This level of agreement is also consistent with previous Johnson–Cook parameter calibration studies based on combined experimental and numerical approaches, where reasonable agreement between validation simulations and tensile or impact responses has likewise been reported [10,11,12,13,14]. The small oscillatory fluctuations retained in the simulated curve are mainly attributed to transient stress-wave effects and contact-induced dynamic response in the explicit SHTB simulation.

Based on the systematic experiments and numerical calibration procedures, Johnson–Cook failure parameters ($D_{1}$ to $D_{4}$) of Cr20Ni80 alloy have been determined. These optimized parameters, which accurately capture both the stress triaxiality dependence and the strain-rate sensitivity of the material’s fracture behavior under complex dynamic loading, are summarized in Table 4.

## 4. Conclusions

In this study, the J-C constitutive model and failure parameters of Cr20Ni80 alloy were calibrated and verified by combining experimental tests and numerical simulations. The main conclusions of this study are as follows:

(1) Calibration of J-C Model Parameters

A reliable set of room-temperature J-C parameters has been successfully established. The constitutive parameters were determined as A = 621.02 MPa, B = 543.20 MPa, n = 0.4564, and C = 0.0141 through quasi-static tensile tests and dynamic SHTB tensile tests. In addition, by combining notch tensile tests with explicit finite element simulations, the failure parameters were calibrated. The corresponding failure parameters were obtained as $D_{1}$ = −0.4300, $D_{2}$ = 2.6405, $D_{3}$ = −0.7055, and $D_{4}$ = −0.0355.

(2) Validation and Application

The calibrated J-C model demonstrated high predictive accuracy under dynamic conditions. For the inverse calibration of $D_{4}$, the experimental and simulated SHTB responses at 1000 s^−1^ and 3000 s^−1^ were compared, and $D_{4}$ was iteratively adjusted until reasonable agreement was obtained. The full calibrated constitutive–failure parameter set was then used to simulate the 2000 s^−1^ test condition for validation. The simulated response under the 2000 s^−1^ validation condition showed good agreement with the experimental curve. The simulation reproduced the main deformation stage and the subsequent stress-drop behavior associated with fracture under the validation condition. These results confirm the validity and reliability of the parameters obtained for simulating the complex manufacturing process and structural forming of Cr20Ni80 alloy.

(3) Limitations and Future Work

The current work was conducted at room temperature; therefore, the temperature-related parameters, especially the thermal softening exponent (m) and the temperature-dependent failure parameter ($D_{5}$), were not calibrated. Future work will focus on high-temperature experiments and repeated notched and dynamic tensile tests to improve the thermal applicability and statistical robustness of the calibrated J-C model. Microstructural observations (e.g., SEM fracture analysis) may also be incorporated to further support the physical interpretation of the calibrated parameters.

## Figures and Tables

**Table 1:** Elemental composition (mass fraction) of Cr20Ni80 alloy, as provided in the manufacturer’s quality certificate according to GB/T 1234-2012 [20].

C	Si	Mn	Cr	P	S	Ni	Al	Fe
0.06	1.41	0.48	20.15	0.011	0.012	Remainder	0.36	0.86

**Table 2:** Relationship between strain rate and dynamic flow stress of Cr20Ni80 alloy.

	1	2	3	4
$\dot{\varepsilon }$	0.0005	1000	2000	3000
${ln\dot{\varepsilon }}^{*}=ln(\dot{\varepsilon }/{\dot{\varepsilon }}_{0})$	0	14.509	15.202	15.607
$\sigma_{eq}∕MPa$	751.153	854.698	938.670	935.913

**Table 3:** Calibrated Johnson–Cook constitutive parameters for Cr20Ni80 alloy.

Parameter	A (MPa)	B (MPa)	n	C
Value	621.02	543.20	0.4564	0.0141

**Table 4:** Failure Model Parameters for Cr20Ni80 alloy.

**Parameters**	$D_{1}$	$D_{2}$	$D_{3}$	$D_{4}$
Values	−0.4300	2.6405	−0.7055	−0.0355

## Data Availability

The data presented in this study are available on request from the corresponding author.
